# Group B *Streptococcus* in the urine in nonpregnant adults: Disease or distraction?

**DOI:** 10.1017/ash.2022.236

**Published:** 2022-08-04

**Authors:** Nicole Mongilardi, Brigid M. Wilson, Taissa A. Bej, Janet M. Briggs, Richard E. Banks, Sunah Song, Robin L. P. Jump, Federico Perez

**Affiliations:** 1 Geriatric Research Education and Clinical Center (GRECC), VA Northeast Ohio Healthcare System, Cleveland, Ohio; 2 Division of Infectious Diseases & HIV Medicine, Department of Medicine, Cleveland Institute for Computational Biology, Cleveland, Ohio; 3 Cleveland Institute for Computational Biology, Cleveland, Ohio; 4 Department of Population and Quantitative Health Sciences at Case Western Reserve University School of Medicine, Cleveland, Ohio

## Abstract

In this large, retrospective cohort study, we used administrative data to evaluate nonpregnant adults with group B *Streptococcus* (GBS) bacteriuria. We found greater all-cause mortality in those with urinary tract infections compared to asymptomatic bacteriuria. Differences in patients’ baseline characteristics and the 1-year mortality rate raise the possibility that provider practices contribute to differences observed.

Invasive infections caused by Group B *Streptococcus* (GBS), including osteomyelitis, skin and soft-tissue infections, and bacteremia without focus, are increasing in the United States.^1,2^ The clinical relevance of noninvasive GBS infections is less characterized, in part because of challenges distinguishing infection from colonization. This includes GBS bacteriuria among nonpregnant adults, in whom urinary colonization rates exceed 20%.^3,4^ We compared people with GBS bacteriuria who were diagnosed and treated for a urinary tract infection (UTI) to those who were not (indicating asymptomatic GBS bacteriuria).

## Methods

### Case ascertainment and clinical characteristics

We conducted a retrospective cohort study of active users of Veterans’ Affairs (VA) health care from January 1, 2008, through December 31, 2017, by accessing data from the VA Corporate Data Warehouse through the VA Informatics and Computing Infrastructure. The Institutional Review Board (IRB) of the VA Northeast Ohio Healthcare System approved the study protocol. Inclusion criteria were VA healthcare users with at least 1 urine culture that was monomicrobial for GBS with a urinalysis (UA) within 3 days of the urine culture. Cases with GBS identified from a culture other than urine or with a polymicrobial urine culture within 90 days were excluded. Only the incident case for each patient was included. Cases with a clinical encounter that included a urine culture associated with an *International Classification of Diseases* (ICD) code for a UTI and an antibiotic prescription within 3 days of the urine culture were classified as UTI cases. Cases with a clinical encounter that included a urine culture and no associated ICD codes for UTI and no antibiotics within 3 days of the urine culture were classified as asymptomatic GBS bacteriuria. Cases with qualifying cultures for both a UTI and asymptomatic GBS bacteriuria were classified as a UTI. Cases with a UTI ICD code and no antibiotic prescription and cases with an antibiotic prescription and no UTI ICD code were unclassifiable and therefore were not included. We analyzed demographics, setting, all-cause mortality at 30 days and 1 year, and the Charlson comorbidity index (CCI) score (determined using ICD codes).^5^ We also assessed antibiotics prescribed for UTI cases.

### Statistical analysis

Clinical and demographic characteristics of patients with UTIs and asymptomatic GBS bacteriuria were compared using *t* tests for continuous variables and χ^2^ tests for categorical variables. We developed multivariable logistic regression models to report odds ratios (ORs) and 95% confidence intervals (CIs) for all-cause mortality at 30 days and 1 year to assess adjusted mortality differences between patients with UTIs and asymptomatic GBS bacteriuria. These models included age, sex, race, ethnicity, CCI, and setting at the time of the urine culture. All statistical analyses were performed using R version 3.5.1 software (R Project for Statistical Computing, Vienna, Austria). All reported *P* values are unadjusted.

## Results

### Patient characteristics

Between 2008 and 2017, we identified 20,906 patients with GBS bacteriuria, of whom 3,223 (15%) were diagnosed and treated for a UTI. Comparing the patients diagnosed and treated for a UTI to those with asymptomatic GBS bacteriuria, the former were older (64.7 ± 15.5 vs 61.2 ± 15.1 years; *P* < .001), had a higher CCI (2.83 ± 2.7 vs 2.14 ± 2.3; *P* < .001), and were more likely to be inpatients (17% vs 5%; *P* < .001). Several chronic conditions, including pulmonary (30% vs 22%; *P* < .001) and renal diseases (20% vs 13%; *P* < .001), were more common among patients diagnosed and treated for a UTI than in those with asymptomatic GBS bacteriuria.

### Antibiotic use

Fluoroquinolones were the most common antibiotic class prescribed for treatment of UTI cases (Table 2). For outpatients, other commonly prescribed antibiotics included trimethoprim-sulfamethoxazole (20%) and nitrofurantoin (9%). Inpatients received β-lactam/β-lactamase inhibitor combinations (31%), extended-spectrum cephalosporins (26%), and penicillins (17%).

**Table 2.** Antibiotic Agents Prescribed for UTI Cases Cultured in the Outpatient and Inpatient Settings^a^

Antibiotic	Outpatient Culture (n = 2,675), No. (%)	Inpatient Culture (n = 548), No. (%)
Fluoroquinolones	1,435 (54)^a^	315 (57)
Trimethoprim/Sulfamethoxazole	548 (20)	81 (15)
Nitrofurantoin	230 (9)	16 (3)
Cephalosporins, extended spectrum	186 (7)	144 (26)
Penicillins	184 (7)	92 (17)
Cephalosporins, 1^st^/2^nd^ generation	158 (6)	56 (10)
β-lactam/β-lactamase inhibitors	146 (5)	172 (31)
Vancomycin	53 (2)	89 (16)
Macrolides	46 (2)	16 (3)
Other^b^	122 (5)	77 (14)

a The percentage refers to the total number of outpatients or inpatients prescribed an antibiotic from the antibiotic classes listed. Some patients received >1 class of antibiotic in the 3 days following the urine culture, so the cumulative percentage exceeds 100%. Some patients were admitted after outpatient culture or discharged after inpatient culture.

b Other includes clindamycin, linezolid, rifaximin, aminoglycosides, and carbapenems.

### Mortality

All-cause mortality was greater among those with who were diagnosed and treated for a UTI compared to those with asymptomatic GBS bacteriuria at 30 days (1.9% vs 0.3%; *P* < .001) and at 1 year (10% vs 4%; *P* < .001). These differences persisted after controlling for age, sex, race, ethnicity, CCI, and inpatient status at time of culture (Supplementary Table 1).

**Table 1.** Characteristics of VA Healthcare System Users with Monomicrobial Urine Cultures Growing GBS, Stratified by Infection Status

Variable	Urinary Tract Infection (n = 3,223)	Asymptomatic Bacteriuria (n = 17,683)	*P* Value
Age, mean y (±SD)	64.7 (±15.5)	61.2 (±15.1)	<.001
Male sex, no. (%)	2,479 (77)	13,143 (74)	<.001
**Race, no. (%)**			<.001
White	2,121 (66)	11,085 (63)	
Black	782 (24)	4,420 (25)	
Other^a^	320 (10)	2,178 (11)	
**Ethnicity, no. (%)**			<.001
Not Hispanic	2,798 (87)	14,723 (83)	
Hispanic	282 (9)	1,927 (11)	
Other^a^	143 (4)	1,033 (6)	
Charlson comorbidity index, mean (±SD)^a^	2.83 (± 2.7)	2.14 (± 2.3)	<.001
**Comorbidities, no. (%)**			
Diabetes mellitus	1,456 (45)	7,386 (42)	<.001
Chronic pulmonary disease	951 (30)	3,955 (22)	<.001
Chronic renal disease	653 (20)	2,299 (13)	<.001
Cancer	575 (18)	2,654 (15)	<.001
Stroke	537 (17)	1,998 (11)	<.001
Chronic heart failure	489 (15)	1,645 (9)	<.001
Peripheral vascular disease	456 (14)	1,759 (10)	<.001
Liver disease	331 (10)	1,533 (9)	<.005
HIV	29 (1)	133 (1)	0.358
**Status at time of urine culture, no. (%)**			<.001
Inpatient	548 (17)	968 (5)	
Outpatient	2,675 (83)	16,715 (95)	
**Mortality, no. (%)**			
30-day all-cause mortality	60 (1.9)	56 (0.3)	<.001
1-year all-cause mortality	325 (10)	701 (4)	<.001

Note. SD, standard deviation; HIV, human immunodeficiency virus.

a American Indian, Alaska Native, Asian, Native Hawaiian or Pacific Islander and unknown; for ethnicity includes unknown.

## Discussion

This national retrospective cohort study detected greater mortality among people with GBS bacteriuria who were diagnosed and treated for a UTI compared to those with asymptomatic GBS bacteriuria. The difference in all-cause mortality at 1 year was unexpected because this extends long beyond the period when sequelae would be expected for UTIs. Baseline differences in the age and chronic medical conditions between patients diagnosed and treated for UTI compared to those with asymptomatic GBS bacteriuria likely contributed to the observed differences in mortality. Although the data cannot entirely rule out the potential pathogenicity of GBS bacteriuria, the differences between patients diagnosed and treated for a GBS UTI compared to those with asymptomatic GBS bacteriuria likely reflects that clinicians are more likely to recommend antibiotics to patients perceived as being at high risk for poor outcomes. Furthermore, the finding that most of the patients who received an antibiotic were issued a fluoroquinolone (not among the first-line agents for GBS infections) suggests that prescribers were not using culture results to guide their decisions, highlighting an opportunity to improve antimicrobial stewardship. To our knowledge, this is the largest study to assess clinical outcomes among nonpregnant individuals with GBS bacteriuria.

Some clinicians conflate pyuria and/or a positive urine culture with UTI, even in the absence of symptoms specific to the genitourinary tract.^6,7^ When considered in context with cognitive biases that influence antibiotic prescribing decisions, the finding that patients with GBS bacteriuria who were older, diabetic, or had chronic pulmonary and renal diseases were more likely to have been prescribed an antibiotic is not surprising.^8^ Increased age, diabetes, cardiovascular disease and renal or urologic pathology are potential risk factors for UTIs due to GBS.^9,10^ Clinical recognition of these risk factors may have also increased the likelihood of requesting urine cultures.

Our study had several limitations. Our cohort comprised VA healthcare users, who are predominantly male and have a high burden of comorbidities, which may limit generalizability to the US population. Although we excluded patients with other cultures that grew GBS, we did not exclude other types of infections. Patients may have received antibiotics for a different indication and were misclassified as having a UTI. Also, our study relied on administrative data and, therefore, providers’ determinations of UTIs rather than clinical signs and symptoms. Thus, we may have overestimated the number of infections because some cases diagnosed and treated by the provider as UTIs may have been asymptomatic GBS bacteriuria. Finally, as discussed above, confirmation bias may have influenced the results; clinicians may have been more likely to obtain urine culture specimens from older, sicker patients and may have been more likely to diagnose and treat such patients for UTI.

In conclusion, our results suggest that GBS bacteriuria is common and that clinicians view only a minority of cases as potential infections. Although patients diagnosed and treated for a GBS UTI had greater all-cause mortality compared to people with asymptomatic GBS bacteriuria, distinctions in patients’ baseline characteristics and 1-year mortality suggest that provider practices contributed to the differences observed.

## Supplementary material

For supplementary material accompanying this paper visit https://doi.org/10.1017/ash.2022.236. click here to view supplementary material

## References

[1] Francois Watkins LK , McGee L , Schrag SJ , et al. Epidemiology of invasive group B streptococcal infections among nonpregnant adults in the United States, 2008–2016. JAMA Intern Med 2019;179:479–488. 30776079 10.1001/jamainternmed.2018.7269 PMC6450309

[2] Jump RLP , Wilson BM , Baechle D , et al. Risk factors and mortality rates associated with invasive group B *Streptococcus* infections among patients in the US Veterans’ Health Administration. JAMA Netw Open 2019;2:e1918324–e1918324. 31880800 10.1001/jamanetworkopen.2019.18324 PMC6991221

[3] van der Mee-Marquet N , Fourny L , Arnault L , et al. Molecular characterization of human-colonizing *Streptococcus agalactiae* strains isolated from throat, skin, anal margin, and genital body sites. J Clin Microbiol 2008;46:2906–2911. 18632904 10.1128/JCM.00421-08 PMC2546740

[4] Edwards MS , Rench MA , Palazzi DL , Baker CJ. Group B streptococcal colonization and serotype-specific immunity in healthy elderly persons. Clin Infect Dis 2005;40:352–357. 15668856 10.1086/426820

[5] Quan H , Sundararajan V , Halfon P , et al. Coding algorithms for defining comorbidities in ICD-9-CM and ICD-10 administrative data. Med Care 2005;43:1130–1139. 16224307 10.1097/01.mlr.0000182534.19832.83

[6] Shively NR , Buehrle DJ , Clancy CJ , Decker BK. Prevalence of inappropriate antibiotic prescribing in primary care clinics within a Veterans’ Affairs healthcare system. Antimicrob Agents Chemother 2018;62:e00337–18. 29967028 10.1128/AAC.00337-18 PMC6105840

[7] Spivak ES , Burk M , Zhang R , et al. Management of bacteriuria in Veterans’ Affairs hospitals. Clin Infect Dis 2017;65:910–917. 28531289 10.1093/cid/cix474

[8] Langford BJ , Daneman N , Leung V , Langford DJ. Cognitive bias: how understanding its impact on antibiotic prescribing decisions can help advance antimicrobial stewardship. JAC-Antimicrob Resist 2020;2:dlaa107. 34223057 10.1093/jacamr/dlaa107 PMC8210114

[9] Ulett KB , Benjamin WH , Zhuo F , et al. Diversity of group B *Streptococcus* serotypes causing urinary tract infection in adults. J Clin Microbiol 2009;47:2055–2060. 19439533 10.1128/JCM.00154-09 PMC2708523

[10] Vigliarolo L , Arias B , Suárez M , et al. Argentinian multicenter study on urinary tract infections due to *Streptococcus agalactiae* in adult patients. J Infect Dev Ctries 2019;13:77–82. 32032027 10.3855/jidc.10503

