# Supplementary material for: Group B Streptococcus in the urine in nonpregnant adults: Disease or distraction?
Source: Antimicrob Steward Healthc Epidemiol. 2022 Aug 4;2(1):e131. doi: 10.1017/ash.2022.236 (PMC9726585; doi:10.1017/ash.2022.236)
Supplement: Supplementary file 1 [file S2732494X22002364sup001.docx]

**Supplementary Table 1.** 30-day and 1-year all-cause Mortality by Infection Status.

|  | **Urinary Tract Infection**  (n = 3223) | **Asymptomatic Bacteriuria**  (n = 17683) | **Adjusted Odds Ratio*** | **95% Confidence Interval** |
| --- | --- | --- | --- | --- |
| Mortality |  |  |  |  |
| 30-day all-cause Mortality | 60 (1.9%) | 56 (0.3%) | 2.37 | 1.58-3.55 |
| 1-year all-cause Mortality | 325 (10%) | 701 (4%) | 1.56 | 1.33-1.82 |

*Adjusted odds ratios estimated in logistic models adjusting for age, sex, race, ethnicity, Charlson comorbidity index, and inpatient at time of culture.
